# Calibration procedure for enhanced mirror artifact removal in full-range optical coherence tomography using passive quadrature demultiplexing

**DOI:** 10.1117/1.JBO.27.11.116006

**Published:** 2022-11-25

**Authors:** Xavier Attendu, Dirk J. Faber, Guy Lamouche, Ton G. van Leeuwen, Caroline Boudoux, Maxime Rivard

**Affiliations:** a Polytechnique Montréal, Centre d’Optique Photonique et Lasers, Department of Engineering Physics, Montréal, Québec, Canada; b University of Amsterdam, Amsterdam University Medical Centers, Department of Biomedical Engineering and Physics, Amsterdam, The Netherlands; c National Research Council Canada, Boucherville, Québec, Canada

**Keywords:** full-range optical coherence tomography, passive quadrature demultiplexing, mirror artifact, calibration

## Abstract

**Significance:**

Passive quadrature demultiplexing allows full-range optical coherence tomography (FR-OCT). However, imperfections in the wavelength- and frequency-response of the demodulation circuits can cause residual mirror artifacts, which hinder high-quality imaging on both sides of zero delay.

**Aim:**

We aim at achieving high mirror artifact extinction by calibrated postprocessing of the FR-OCT signal.

**Approach:**

We propose a mathematical framework for the origin of the residual mirror peaks as well as a protocol allowing the precise measurement and correction of the associated errors directly from mirror measurements.

**Results:**

We demonstrate high extinction of the mirror artifact over the entire imaging range, as well as an assessment of the method’s robustness to time and experimental conditions. We also provide a detailed description of the practical implementation of the method to ensure optimal reproducibility.

**Conclusion:**

The proposed method is simple to implement and produces high mirror artifact extinction. This may encourage the adoption of FR-OCT in clinical and industrial systems or loosen the performance requirements on the optical demodulation circuit, as the imperfections can be handled in postprocessing.

## 1 Introduction

In its standard implementation, Fourier-domain optical coherence tomography (FD-OCT) suffers from the *complex conjugate* or *mirror artifact*, which prevents simultaneous imaging on both sides of the zero-delay plane.^1^ In addition to reducing the overall imaging range, this configuration requires the sample to be located at a certain distance from the zero-delay plane, which may lead to a reduction in sensitivity due to spectral roll-off.^2^ Several methods have been proposed to overcome these limitations and enable *full-range* optical coherence tomography (FR-OCT), which allows artifact-free imaging on both sides of the zero-delay plane. These methods can be subdivided into four categories: frequency modulation techniques, Talbot bands OCT, phase-shifting interferometry, and passive quadrature demultiplexing methods. The advantages and limitations of each option are briefly discussed below.

The frequency modulation approach (also called heterodyne OCT) relies on the use of active optical components such as acousto- and electro-optic modulators (AOM and EOM, respectively) to superimpose a modulation on top of interference signal in optical frequency-domain imaging.^2^^,^^3^ This modulation causes the interference signal associated with a null spatial offset to be shifted to a nonzero frequency, allowing imaging with positive and negative spatial offsets. Frequency modulation provides excellent extinction of the complex conjugate (≥60  dB)^2^ as well as improved signal-to-noise ratio (SNR) due to the elimination of low-frequency noise.^3^ Here, we define the mirror artifact extinction as the ratio of the original mirror signal over the attenuated one. It is worth noting that an extinction of 60 dB is considered excellent as OCT imaging of biological tissue rarely presents such a broad dynamic range. Indeed, if the extinction exceeds the SNR of the signal (i.e., peak height relative to the noise floor), the mirror artifact will be lowered below the noise floor and effectively removed. Although frequency modulation with active optical devices provides excellent performance, it also presents certain drawbacks, including experimental complexity, increased costs, and limitations of the optical bandwidth due to the AOM/EOM. A frequency modulation, and the corresponding shift of the peak sensitivity, can also be achieved without active components through the phenomenon of coherence revival. This effect occurs when using swept lasers presenting multiple longitudinal modes,^4^ in which case interference may occur for optical path length differences around integer numbers of laser cavity lengths. Coherence revival OCT is particularly appealing because it provides the same benefits as active frequency modulation without the additional cost or bandwidth limitations.^5^^–^^7^ However, it is limited to specific laser sources which present this effect.^4^^,^^5^ All frequency modulation techniques also have the disadvantage of enhanced bandwidth requirements for the detectors and high-speed digitizers as interference is captured at higher frequencies. Finally, such methods are limited to swept-source OCT (SS-OCT) systems and are incompatible with (spectrometer-based) spectral-domain systems (SD-OCT).

By contrast, Talbot bands OCT is a full-range method specific to SD-OCT systems. This approach utilizes a different interferometer configuration where the sample and reference beams are spatially separated and do not interfere until the detection plane in the spectrometer. By changing the spatial offset and beam characteristics at the dispersive element, the sensitivity profile can be shifted away from a null optical path difference and tailored in shape.^8^^,^^9^ In specific configurations, this can lead to the total extinction of the mirror artifact,^8^ albeit at the cost of overall sensitivity of the system.^10^^,^^11^

Another approach for full-range OCT consists of using phase-shifting interferometry to reconstruct the complex interference signal (i.e., both the phase and amplitude of the interference fringes) from two or more interference spectra acquired sequentially.^1^^,^^12^^,^^13^ Options to obtain a phase shift between the acquisitions include piezoelectric transducers (PZT) mounted on the reference mirror^13^^–^^15^ and electro-optic phase modulators.^16^^,^^17^ Such methods, however, require multiple acquisitions per A-line, thereby reducing the overall acquisition speed and increasing their sensitivity to sample motion and the system’s overall phase stability. A variant of the phase-stepping approach, called *BM-scanning*, removes the requirement for multiple acquisitions by introducing the phase shift across A-lines within a B-scan. The complex signal can then be recovered by performing a Hilbert transform (HT) across the spatial dimension prior to applying the usual Fourier transform (FT).^18^^–^^23^ Phase-shifting methods, in general, have the added benefit that they can be implemented in both types of Fourier domain OCT (FD-OCT) systems. However, to the best of our knowledge, none of the phase-shifting approaches have achieved extinction of the complex conjugate equivalent to that obtained with frequency modulation, and are usually limited to ≤40  dB.^13^^,^^14^^,^^17^^,^^23^

The last group of FR-OCT methods uses optical systems to passively separate and simultaneously measure the complex interference signal’s real and imaginary components. This can be accomplished using polarization optics^24^^,^^25^ or alternative interferometer configurations with N×N fiber couplers, with N≥3.^26^^–^^28^ In both instances, however, the separation of the signal components must be stable, uniform across the complete spectral bandwidth, and precisely known. This typically requires highly accurate and time-consuming calibrations or sophisticated and expensive optical components. Given these complex specifications, passive demultiplexing commonly does not achieve high extinction ratios. It is usually also limited to extinction ratios ≤40  dB.^24^^–^^28^ Like phase-shifting methods, passive demultiplexing is compatible with both FD-OCT techniques. However, the passive approach is usually only applied to SS-OCT systems as it requires two detection channels. In the case of SD-OCT, two spectrometers would be required, significantly increasing the system’s cost. Finally, the simultaneous measurement of both the real and imaginary signal components allows for maintaining high acquisition speeds and makes passive demultiplexing insensitive to sample motion.

As such, no method is dominant: each has drawbacks in terms of cost, complexity, stability, or overall performance. In this paper, we propose a calibration protocol for passive demultiplexing, which may address some shortcomings mentioned in the previous paragraph. We propose a simple and straightforward procedure to experimentally assess and correct the relative differences in the two measurement channels, including the chromatic or spectral effects as well as the *RF-errors* associated with detection and acquisition electronics.^29^ The proposed method relies only on measuring the interference signal from a mirror at different depth locations and can be performed directly on the system without any modifications other than adding the optical quadrature demultiplexing circuit. We first describe the underlying theoretical framework, then demonstrate the proposed method on an SS-OCT system with a polarization-based demultiplexing scheme. However, it is important to note that the method may be generalized to other passive quadrature demultiplexing schemes in FD-OCT.

## 2 Theory–Complex Signal Reconstruction

The aim of passive quadrature demultiplexing is the reconstruction of the complex interference signal, $\tilde{S}$, which lifts the ambiguity between the positive and negative delays after FT or, in other words, removes the mirror artifact. This reconstruction requires the simultaneous measurement of the real and imaginary parts of the interference signal, also called the real ($S_{R}$) and quadrature ($S_{Q}$) signals. In principle, these two signals are identical except for a π/2 phase shift at all wavelengths and axial positions. An ideal demodulation circuit allows the direct measurement of these signals in two output channels, which can then be used to reconstruct the complex signal following:

$$\tilde{S}=S_{R}+i\cdot S_{Q}.$$ (1)

In real applications, however, there exist minor deviations in the phase and amplitude of the two measured signals, which results in residual mirror peaks (see Appendix A for more details). The accuracy in amplitude- and phase-matching required to achieve mirror artifact extinction ≥60  dB cannot be achieved with physical components and necessitates the measured signals to be corrected in postprocessing. This work outlines a method to perform the necessary adjustments to the measured signals to achieve high mirror peak extinction. We begin by deriving expressions for the OCT signal, which encompasses the effects of the demodulation circuit. We show that, for each channel, the combined effect of all components in the passive demodulation scheme can be simplified down to two phasors: one wavelength-dependent and one depth-dependent. Finally, we propose a method to measure these phasors and correct the measured signals accordingly.

In Eq. (1) and throughout this manuscript, we use variables with a tilde symbol to denote complex-valued vectors. Variables without the tilde symbol refer to the real component of their complex counterpart (e.g., $S=R\{\tilde{S}\}$). While the mathematical derivations presented here utilize complex signals, all measurement data consists of real values. The *measured* complex signals are the analytic representation of the real-valued signals, obtained via an adaptation of the HT, as discussed in Sec. 3.2. These measured complex signals should not be confused with the *reconstructed* complex signal.

### 2.1 Chromatic Errors

The complex signal measured at the n’th output of the demodulation circuit can be described as

$${\overset{˜}{S}}_{n}(k)=\underset{\mathrm{DC}\text{ terms}}{\underbrace{I_{R}T_{Rn}+\underset{m}{\sum }T_{Sn}R_{m}I_{S}}}+\underset{\text{interference term}}{\underbrace{\underset{m}{\sum }2\sqrt{T_{Rn}T_{Sn}R_{m}I_{R}I_{S}}e^{i(\theta_{m}+\phi_{n})}}},$$ (2)

where n=1−4, $I_{S}$, and $I_{R}$ are the intensities in the sample and reference arms, respectively, $T_{Sn}$ and $T_{Rn}$ are the transmission efficiencies from the sample and reference arms, respectively, $R_{m}$ is the intensity reflectivity of the m’th reflector in the sample arm, $\theta_{m}$ is the phase ramp associated to the position of the reflector, $\Delta z_{m}$, and is equal to $2k\Delta z_{m}$, and $\phi_{n}$ is the phase offset attributed to the n’th output port. All the above variables (with the exception of $\Delta z_{m}$) are functions of the wavenumber, k. Assuming that the DC components are removed through balanced detection and background removal, the balanced interference signals, ${\overset{˜}{S}}_{I,\mathrm{int.}}(k)$ and ${\tilde{S}}_{II,\mathrm{int}.}(k)$, can be isolated, as

$${\overset{˜}{S}}_{I,\mathrm{int}.}(k)=\underset{m}{\sum }2\sqrt{R_{m}}e^{i\theta_{m}}\cdot [\sqrt{T_{R1}T_{S1}I_{R}I_{S}}e^{i\phi_{1}}-\sqrt{T_{R3}T_{S3}I_{R}I_{S}}e^{i\phi_{3}}]\;{\overset{˜}{S}}_{II,\mathrm{int}.}(k)=\underset{m}{\sum }2\sqrt{R_{m}}e^{i\theta_{m}}\cdot [\sqrt{T_{R2}T_{S2}I_{R}I_{S}}e^{i\phi_{2}}-\sqrt{T_{R4}T_{S4}I_{R}I_{S}}e^{i\phi_{4}}].$$ (3)

Here, we assume that the autocorrelation terms, suppressed through balanced detection, are negligible. The validity of this assumption is particularly important in the context of FR-OCT as autocorrelation artifacts usually occur close to zero-delay, an area now part of the useful imaging range. While autocorrelation artifacts from biological tissue tend to be negligible, multiple specular reflections from optical components in the interferometer arms can lead to more significant ones. Fortunately, such specular reflections can be mitigated through the proper choice of optics and antireflection coatings. To lighten the mathematical notation, the subscript int. is dropped from Eq. (3), and the measured signals ${\tilde{S}}_{I}$ and ${\tilde{S}}_{II}$ will refer solely to the interference signal. Equation (3) is simplified by grouping the transmission and intensity variables, extracting from the summation the terms that are not depth-dependent, and using the fact that the sum of two phasors can be rewritten as a single one. This results in a condensed expression for the complex, balanced signals

$${\overset{˜}{S}}_{I}(k)=A_{I}(k)e^{i\Omega_{I}(k)}\underset{m}{\sum }C_{m}e^{i\theta_{m}}\;{\overset{˜}{S}}_{II}(k)=A_{II}(k)e^{i\Omega_{II}(k)}\underset{m}{\sum }C_{m}e^{i\theta_{m}},$$ (4)

where $A_{I/II}$ and $\Omega_{I/II}$ are the wavelength-dependent terms describing the amplitude and phase of the term in square brackets in Eq. (3), and $C_{m}$ is proportional to the amplitude reflectivity ($r_{m}=\sqrt{R_{m}}$) of the reflector at depth $\Delta z_{m}$. The terms in the summation represent the interference signal unaltered by the demodulation circuit, which is identical in both channels. The two complex signals ${\tilde{S}}_{I}$ and ${\tilde{S}}_{II}$ will only differ from system contributions allowing for a definition of an amplitude ratio $\beta (k)=A_{I}/A_{II}$ and an overall phase offset $\Delta \phi (k)=\Omega_{II}-\Omega_{I}$.

For an ideal demodulation system with perfectly balanced power separation (all $T_{Sn}$ and $T_{Rn}$ being equal) as well as perfect phase characteristics [$\phi_{n}=(n-1)\cdot \pi /2$], the amplitudes of ${\tilde{S}}_{I}$ and ${\tilde{S}}_{II}$ are equal and the phase offset between the two channels is exactly equal to π/2. As such, the measured signals are in perfect quadrature, such that $S_{R}=R\{{\overset{˜}{S}}_{I}\}=S_{I}$ and $S_{Q}=R\{{\tilde{S}}_{II}\}=S_{II}$. The measured signals can, therefore, directly be used in Eq. (1) to reconstruct the complex signal. However, in the non-ideal case, the perfect quadrature must first be reconstructed following:^26^^,^^28^

$$S_{Q}(k)=\frac{\beta (k)\cdot S_{II}(k)-\cos\;\Delta \phi (k)\cdot S_{I}(k)}{\sin\;\Delta \phi (k)},$$ (5)

while still using $S_{R}=S_{I}$. A complete derivation of this equation is provided in Appendix B. When computing the values of β and Δϕ, it is crucial to avoid interchanging the terms from channels I and II in the amplitude ratio and the phase difference. While the definition of channels I and II is arbitrary, it is important to remain consistent once a label has been assigned to each detection channel.

Equation (5) requires that β(k) and Δϕ(k) be known. In the past, these values have been evaluated by measuring the transmission and phase properties of the demodulation circuit separately^26^^,^^28^ or through optimization routines that converge on the values producing the highest extinction of the mirror artifact.^29^ Given Eq. (4), we propose that β(k) and Δϕ(k) can be determined directly from a simple mirror measurement. Indeed, the phase and amplitude of the two real measured signals can be recovered using a HT. Amplitude vectors can then be divided by one another to obtain β, while phase vectors can be subtracted to obtain Δϕ. It is worth mentioning that computing the amplitude and phase of the measured signals does not allow the extraction of the absolute transmission and phase characteristics of the demodulation circuit [i.e., variables $T_{\mathrm{Rn}}$, $T_{\mathrm{Sn}}$, and $\phi_{n}$ in Eq. (2)]. Fortunately, the absolute values are not necessary for the proposed method.

### 2.2 RF-Errors

Siddiqui et al.^29^ identified radio frequency-errors (RF): a second source of errors in the reconstruction of the quadrature signal. These errors are specific to swept-source OCT as they originate in the temporal encoding of the spectral information and all associated distortions. Such RF errors include fiber or electrical cable length differences between the two channels, causing one signal to be delayed relative to the other. Other variations include the frequency response of all electronic components in the detection circuit, such as the detectors, filters, and the data acquisition card. Each of these effects will generate frequency and, therefore, depth-dependent amplitude and phase variations, which will affect the computed values for β and Δϕ. It is, therefore, necessary to expand Eqs. (2)–(5) to incorporate the axial dependence. Failing to account for these variations will result in calibration vectors only valid at the specific depth at which they were computed. It is important to note that the terms RF-errors and spatial errors variations are used interchangeably throughout this manuscript. In the time-domain (or equivalently, in k-space), the impact of the various electronic components can be described as a series of convolutions of the original signal by the impulse responses, $h_{i,j}(t)$, of each element, where the first subscript represents the channels (I or II) and the second subscript the element in the series of electronic components:

$${\overset{˜}{S}}_{I}^{\prime }(t)=((({\overset{˜}{S}}_{I}(t))*h_{I,1}(t))*h_{I,2}(t))*\ldots \;{\overset{˜}{S}}_{II}^{\prime }(t)=((({\overset{˜}{S}}_{II}(t))*h_{II,1}(t))*h_{II,2}(t))*\ldots$$ (6)

Using the convolution theorem, it is convenient to describe the signal in Fourier domain (i.e., in frequency-space), where the chain of convolutions becomes a simple multiplication of the elements’ transfer functions, ${\tilde{H}}_{i}(f)=F_{t}\{h_{i}(t)\}$, as described below:

$$F_{t}\{{\overset{˜}{S}}_{I}^{\prime }\}(f)=F_{t}\{{\overset{˜}{S}}_{I}\}(f)\cdot {\overset{˜}{H}}_{I,1}(f)\cdot {\overset{˜}{H}}_{I,2}(f)\cdot \ldots \;F_{t}\{{\overset{˜}{S}}_{II}^{\prime }\}(f)=F_{t}\{{\overset{˜}{S}}_{II}\}(f)\cdot {\overset{˜}{H}}_{II,1}(f)\cdot {\overset{˜}{H}}_{II,2}(f)\cdot \ldots$$ (7)

In the ideal case of a k-linear sweep, there exists a linear correspondence between time (t) and wavenumber (k), resulting in an equivalent correspondence between temporal frequency (f) and spatial position of the reflector (z). If the wavelength sweep is not linear-in-wavenumber, the interference signal must be sampled nonlinearly with a k-clock or resampled through interpolation in postprocessing. Depending on the k-clock or resampling function, this may lead to a nonlinear relationship between the transfer functions expressed in frequency space, ${\tilde{H}}_{i,j}(f)$, and in z-space, ${\tilde{H}}_{i,j}(z)$. Fortunately, the nature of this relationship and the exact expression for each transfer function expressed in z-space are not important to the proposed method. Indeed, the individual transfer functions, ${\tilde{H}}_{i,j}(z)$, can be grouped into a single phasor accounting for both amplitude and phase variations

$$F_{k}\{{\overset{˜}{S}}_{I}^{\prime }(k)\}=F_{k}\{{\overset{˜}{S}}_{I}(k)\}\cdot H_{I,1}(z)\cdot H_{I,2}(z)\cdot \ldots =F_{k}\{{\overset{˜}{S}}_{I}(k)\}\cdot \alpha_{I}(z)e^{i\omega_{I}(z)}\;F_{k}\{{\overset{˜}{S}}_{II}^{\prime }(k)\}=F_{k}\{{\overset{˜}{S}}_{II}(k)\}\cdot H_{II,1}(z)\cdot H_{II,2}(z)\cdot \ldots =F_{k}\{{\overset{˜}{S}}_{II}(k)\}\cdot \alpha_{II}(z)e^{i\omega_{II}(z)},$$ (8)

where $\alpha_{i}(z)$ and $\omega_{i}(z)$ are the depth-dependent amplitude and phase variations for channel i, respectively. The step from Eq. (7) to Eq. (8) (i.e., the conversion from time to k-space) is important because it implies that the method is valid for k-linear and non-k-linear wavelength sweeps using a k-clock or resampled in postprocessing. It is important to note that all subsequent FTs operate between wavenumber- and z-space. Now, let us consider the case of a single perfect reflector in the sample arm (i.e., a mirror). In this case, the summation in the expression of the complex, balanced signals [Eq. (4)] can be removed, and a single complex exponential remains. The FT of this signal becomes a complex peak, ${\tilde{\gamma }}_{i}(z-\Delta z)$, centered around the position of the reflector, Δz. Inserting this expression of the peak into Eq. (8), we obtain

$$F\{{\overset{˜}{S}}_{I}^{\prime }\}={\overset{˜}{\gamma }}_{I}(z-\Delta z)\cdot \alpha_{I}(z)e^{i\omega_{I}(z)},F\{{\overset{˜}{S}}_{II}^{\prime }\}={\overset{˜}{\gamma }}_{II}(z-\Delta z)\cdot \alpha_{II}(z)e^{i\omega_{II}(z)},$$ (9)

which is simply the interference signal multiplied by the system’s transfer function. Due to the Dirac-like nature of the axial point-spread function (PSF), the primary contribution of the transfer function will be at the depth corresponding to the center of the interference peak. Operating under this assumption that the signal peak is Dirac-like (i.e., very narrow and negligible everywhere except at the center of the peak), we can approximate the signal in each channel as

$${\overset{˜}{S}}_{I}^{\prime }(k)=A_{I}(k)e^{i\Omega_{I}(k)}Ce^{i\theta }\cdot \alpha_{I}(\Delta z)e^{i\omega_{I}(\Delta z)}\;{\overset{˜}{S}}_{II}^{\prime }(k)=A_{II}(k)e^{i\Omega_{II}(k)}Ce^{i\theta }\cdot \alpha_{II}(\Delta z)e^{i\omega_{II}(\Delta z)}.$$ (10)

Qualitatively, we can understand this approximation as the fact that the interference signal of a single reflector is a pure (single-frequency) sinusoid. Therefore, it will only experience a constant amplitude modulation and phase delay due to the system’s frequency response. This approximation is crucial to the proposed method as it enables the direct measurement of the depth-dependence of both β and Δϕ. The initial expressions for the amplitude ratio and the phase offset between the two channels can now be adapted to incorporate the z-dependence

$$\beta (k,z)=\frac{\left\|{\overset{˜}{S}}_{I}^{\prime }\right\|}{\left\|{\overset{˜}{S}}_{II}^{\prime }\right\|}=\underset{\beta_{k}}{\underbrace{\frac{A_{I}(k)}{A_{II}(k)}}}\cdot \underset{\beta_{z}}{\underbrace{\frac{\alpha_{I}(z)}{\alpha_{II}(z)}}};$$ (11)

$$\Delta \phi (k,z)=∡{\overset{˜}{S}}_{II}^{\prime }-∡{\overset{˜}{S}}_{I}^{\prime }=\underset{\Delta \phi_{k}}{\underbrace{\left[\Omega_{II}(k)-\Omega_{I}(k)\right]}}+\underset{\Delta \phi_{z}}{\underbrace{\left[\omega_{II}(z)-\omega_{I}(z)\right]}}.$$ (12)

Obtaining the parameters β and Δϕ from multiple mirror measurements located at different axial positions can simultaneously inform us on the chromatic and spatial (RF) variations of the demodulation circuit. The spatial variations between the two signals can then be corrected by simply multiplying ${\tilde{S}}_{II}^{\prime \prime }$ by the phasor describing the relative difference between the transfer functions

$${\overset{˜}{S}}_{II}^{\prime \prime }=F^{-1}\{F\{{\overset{˜}{S}}_{II}^{\prime }\}\cdot \beta_{z}\cdot e^{-i\Delta \phi_{z}}\}.$$ (13)

The corrected signal, ${\overset{˜}{S}}_{II}^{\prime \prime }$, shares its axial dependence with ${\tilde{S}}_{I}^{\prime }$. As such, the spatial dependence will be removed from β and Δϕ, and a unique set of chromatic correction vectors can be defined for all axial positions ($\beta_{k}$ and $\Delta \phi_{k}$). Finally, the perfect quadrature component, $S_{Q}^{\prime }$, which accounts for both spatial and chromatic effects, can be reconstructed using Eq. (5), with $S_{I}$ and $S_{II}$ replaced with $S_{I}^{\prime }$ and $S_{II}^{\prime \prime }$

$$S_{Q}^{\prime }=\frac{\beta_{k}\cdot S_{II}^{\prime \prime }-\cos\;\Delta \phi_{k}\cdot S_{I}^{\prime }}{\sin\;\Delta \phi_{k}},$$ (14)

where $S_{I}^{\prime }$ corresponds to the real-valued, unaltered signal in channel I, while $S_{II}^{\prime \prime }$ is the real, corrected signal in channel II, derived from the measured signal $S_{II}^{\prime }$ using Eq. (13). This corrected quadrature signal can then be, in turn, used in Eq. (1) to reconstruct the corrected complex interference signal following:

$$\overset{˜}{S}=S_{I}^{\prime }+i\cdot S_{Q}^{\prime }.$$ (15)

Finally, it should be noted that the calibration vectors extracted by this protocol will only be valid for a given set of acquisition parameters, such as the A-line rate, number of samples per A-line, sampling rate, spectral bandwidth, etc. Varying any of these parameters affects the pixel-to-wavelength correspondence within an A-line as well as characteristics of the imaging range, such as physical pixel size and total imaging range. As such, previously obtained correction vectors will not be compatible with the signal acquired in the new configuration.

## 3 Methods

### 3.1 FR-OCT System Description

**Fig. 1:**
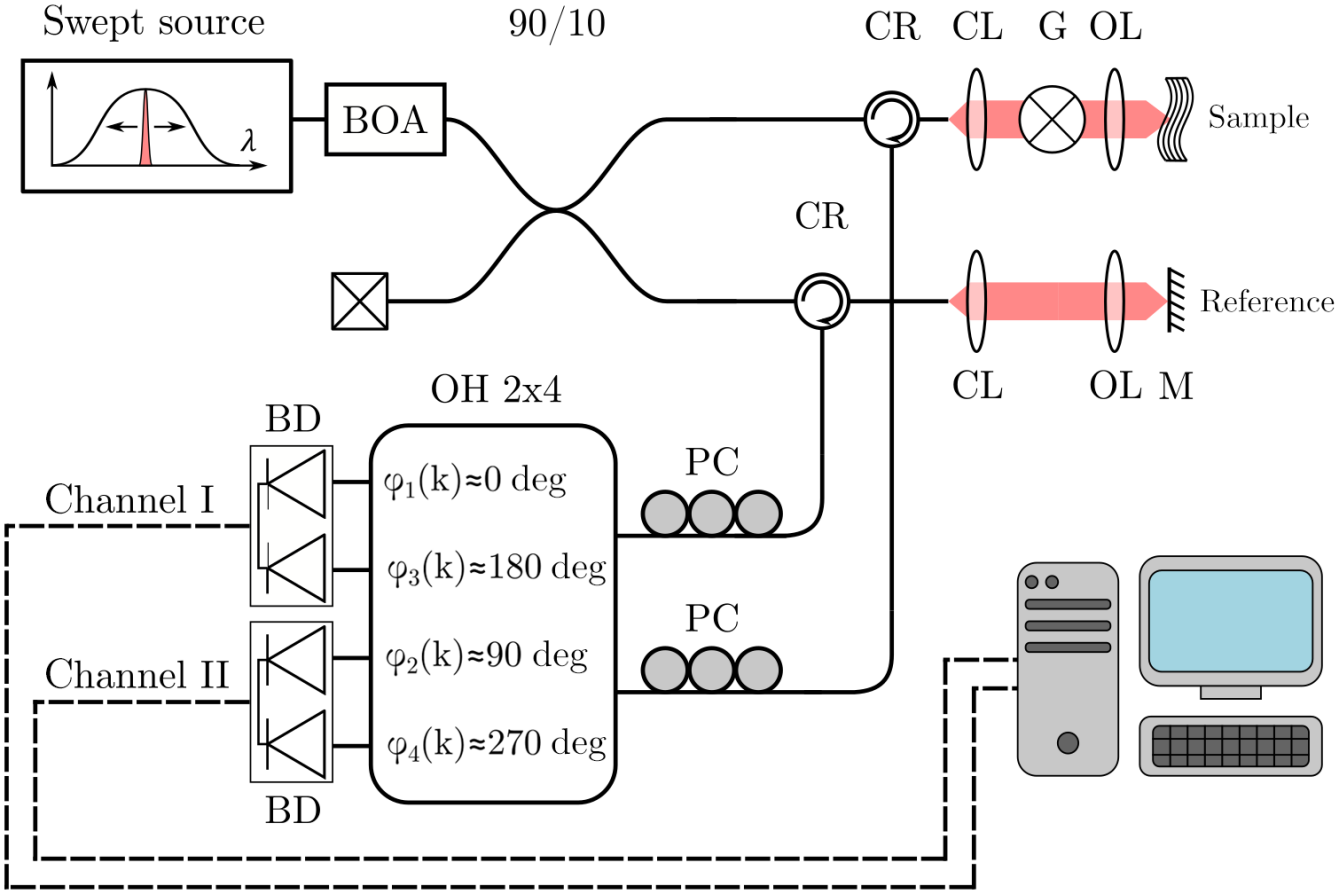
Experimental setup: **CR** fiber circulator, **CL** collimator lens, **G** galvo-scanner, **OL** objective lens, **M** mirror, **PC** polarization controller, **OH** optical hybrid, **BD** dual balanced detector, **BOA** booster optical amplifier. Solid lines are optical fibers and dashed lines are electrical cables. The detail of the internal components of the optical hybrid can be found in the component’s datasheet.

The proposed calibration method was experimentally validated on the setup depicted in Fig. 1, a swept-source OCT system centered at 1550 nm. We utilize a linear-in-wavenumber swept laser source (Insight Akinetic Swept Laser, Insight Photonic Solutions, USA) with a spectral bandwidth of 80 nm, variable-sweep rate, and an average output power of 10 mW. The source output was further increased by an amplifier (BOA, Thorlabs, USA) to 75 mW. Laser light is separated into the sample and reference arms using a 90/10 fiber optic coupler. Light backscattered in both arms is redirected toward an interferometer using fiber circulators. In this work, the selected interferometer design is a Mach–Zehnder terminated with a 2×4 optical hybrid (COH24, Kylia, France). The 2×4 optical hybrid (OH) performs both the interferometric recombination of the sample and reference signals, as well as the quadrature demultiplexing. Demultiplexing in the 2×4 OH is performed using a free-space, polarization-based scheme to generate four output signals with π/2 offsets. The π/2 increments in phase offset are practical as signals of opposite sign (π phase shift) can be measured with balanced detection, thereby removing DC signals and common noise. While we demonstrate the method on this setup, the theoretical framework may be extended to other polarization-based methods as well as N×N fused or integrated optical hybrids, provided N>2. The output signals of the two balanced detectors (BDP-1, Insight Photonic Solutions) are connected to a dual-channel, high-speed digitizer for acquisition of the measurement data (ATS9360, Alazar Technologies, Canada). Measurements were performed using in-house LabView Software (LabView, National Instruments, USA), and data analysis was carried out in MATLAB (MATLAB, Mathworks, USA). All experimental data and processing scripts are available in the Supplementary Material, in Code 1 and Dataset 1 specifically (see Refs. 30 and 31).

### 3.2 Complex Signal Reconstruction – Practical Implementation

**Fig. 2:**
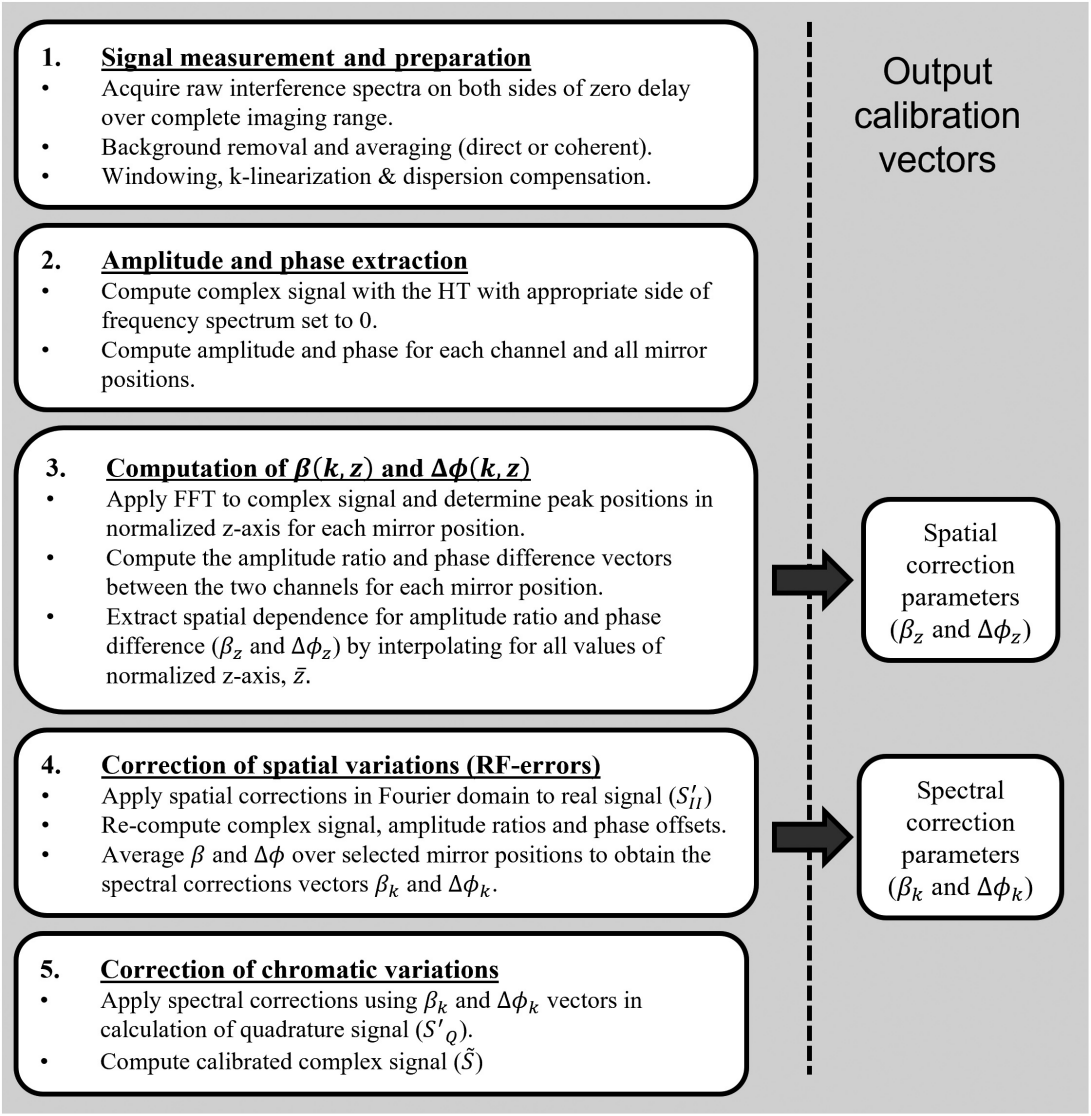
Flowchart of the calibration process. The box numbers correspond to the subsection numbers in Sec. 3.2 of this manuscript.

The theory outlined in the previous section predicts that it is possible to reconstruct the perfect quadrature signal, thereby achieving complete extinction of the mirror artifact. In practice, however, several factors, such as system noise, mechanical or thermal instabilities, numerical errors or approximations, and measurement errors, affect the end result. As such, given the extremely high precision requirements necessary to achieve extinctions ≥60  dB, enhanced attention to detail is necessary during data acquisition and processing. Figure 2 presents the practical steps that should be performed in order to correctly implement the proposed method and achieve optimal mirror artifact extinction.

#### 3.2.1 Signal measurement and preparation

All measurements for the calibration procedure are performed using a mirror as the reflector in the sample arm. As explained in Sec. 2.2, each mirror position provides only the relative difference in the frequency response of the two channels at the position at which the measurement is performed. As such, it is necessary to perform measurements across the entire intended imaging range (i.e., on both sides of zero delay). Additionally, these measurements couple well with calibration measurements necessary for k-linearization and dispersion compensation.^32^ The total number of measurements at different axial positions should be sufficient to capture variations in the transfer function. A larger number of measurement points will also enhance the robustness of the numerical fits performed in this process. The frequency response of the various electronic components (detectors, filters, or data acquisition cards), usually specified in datasheets, may provide insight into the expected behavior of the overall transfer function. Finally, the exact spacing between measurements is not critical, as the method does not require knowledge of the absolute axial position, only the normalized one, which can be obtained by fitting the processed peak. Nonuniform sampling in depth (e.g., denser around depths with expected variations in the transfer function) is also acceptable and even beneficial as it allows for a more accurate interpolation. For example, when using AC-coupled detectors, substantial variations can be expected around DC.

Although measurements can be performed by adjusting the length of either arm of the interferometer, it is usually simpler to alter the length of the reference arm. Extending the collimated segment of the reference arm rather than moving the sample mirror leaves the sample mirror in the focal plane of the objective lens. This will ensure a higher and more stable signal intensity for all measured positions. It is interesting to note that the protocol can function when moving the sample mirror, as both channels will experience the same signal fluctuations due to the confocal beam parameter. However, measurements far outside the Rayleigh range will have a lower SNR, which may induce errors in calculating the correction vectors.

The proposed method relies on using the HT to compute the complex analytical signal, from which phase and amplitude information can be extracted. This transform is highly sensitive to DC offsets. Proper background removal should be performed on all measurements, where the background signal should be measured once for each mirror position. Further DC removal can be achieved by subtracting the mean from the signal. In standard OCT systems, high-pass filtering may be performed to remove the residual low-frequency signal. This is not advised in FR-OCT as it will result in an attenuated band around zero-delay (i.e., in the middle of the measurement range).

In this work, phase and amplitude information is extracted directly from calibration measurements. The SNRs associated with these values are, therefore, directly proportional to that of the measurements. As such, the proposed method cannot result in extinction ratios exceeding the SNR of the calibration measurement (see Appendix A for more detail). Consequently, performing coherent averaging of multiple A-lines is highly recommended to maintain peak amplitude while lowering the noise floor. Coherent averaging is achieved by correcting the phase drift and jitter that may occur between A-lines in SS-OCT prior to computing the mean interference signal. In this work, we followed the averaging method outlined in Ref. 33 A crucial point is that, for each A-line, the same phase correction must be applied to both channels of the demodulation circuit to preserve their relative phase.^29^ The proposed method will fail if coherent averaging is applied to the two channels independently of one another. Coherent averaging is also necessary for this instance to enable visualization of attenuation >60  dB, or else the mirror artifact is hidden below the noise floor.

Finally, the calibration measurements must be properly k-linearized, either through hardware methods or through numerical resampling, prior to their use in the quadrature correction. Nonlinear k-sampling may introduce phase and amplitude variations comprising mixed terms that depend on both wavenumber and depth. In such a scenario, it would no longer be possible to separate the chromatic and axial contributions, as depicted in Eqs. (11) and (12). This would also undermine the approximation in Eq. (10) as non-k-linear sampling results in broader and, more importantly, a depth-dependent axial PSF. However, numerical resampling may be performed prior to implementing the proposed method without any impact on overall performance. It is not mandatory to compensate for dispersion on the calibration measurement prior to using them in the quadrature correction as the dispersion mismatch will result in a depth-independent, nonlinear phase term in the interference signal^32^ that is identical in both channels of the demodulation circuit. However, performing dispersion compensation is recommended as it will yield calibration measurements with higher SNR. Furthermore, dispersion-compensated peaks will be narrower, supporting the approximation required for the validity of Eq. (10). It is also interesting to note that phase stability is not a prerequisite for the successful implementation of this method. Indeed, phase instabilities in the system will be equally represented in both detection channels and, therefore, will not affect the corrections.

#### 3.2.2 Computing analytical signal and extracting amplitude and phase

We compute the complex analytical signal for each mirror position and channel, typically using the HT. Many different numerical implementations of the HT exist, which perform a different number of operations. Strictly speaking, the HT should return an identical signal phase-shifted by π/2. The analytical signal can then be reconstructed using the input signal as the real component and the HT as the imaginary part. However, many implementations of the HT (e.g., in MATLAB) directly return the analytic representation of the input signal by applying an FT on the input signal, setting all negative frequency components to 0, and then performing the inverse FT. This approach, therefore, assumes that the true signal peak is always located on the positive side of zero-delay. For the calibration, this should be altered for peaks measured on the negative side of zero-delay, such that the analytical signal has all positive frequency components set to zero. This manipulation is required during the calibration only, when the position of the mirror is known, and not during subsequent imaging. Once the complex analytical signal is obtained, the amplitude and phase at each sample point can be obtained using standard identities. Care should be taken to employ the appropriate inverse tangent for phase extraction (typically called *atan2*) as well as proper angle unwrapping to avoid discontinuities in the phase vector. Such discontinuities will cause significant errors in subsequent fitting and cause the method to fail.

#### 3.2.3 Computing β(k,z) and Δϕ(k,z)

The correction vectors β(k,z) and Δϕ(k,z) are defined as a function of wavenumber, k, and axial position, z. In the spatial dimension, however, they are only sampled at the positions at which mirror measurements are performed, $\Delta z_{m}$. Interpolation is, therefore, necessary to reconstruct the correction vectors at all axial positions. First, to establish the positions at which measurements were obtained, it is necessary to evaluate the magnitude of the FT of the interference signal for each channel. This can be performed on the complex analytical signals computed in the previous step. When the analytical signals are properly generated, peaks should appear only on the correct side of zero-delay. Although the measured axial position may be used, extracting the peak positions directly from the measured signals is recommended, as this may avoid experimental errors and simplify the experimental protocol. Gaussian fitting (or a simple search for the maximum value) may be used to find the exact position of the peak. Furthermore, rather than absolute values in millimeters, it is convenient to use a normalized z-axis, defined relative to the number of samples, N, in the spectral interferogram as follows:

$$\overset{¯}{z}=\{\begin{array}{ll} \left[-\frac{\pi }{2};\frac{\pi }{2}-\frac{\pi }{N}\right] & \text{for }N\text{ even}\\ \left[-\frac{\pi }{2};\frac{\pi }{2}\right] & \text{for }N\text{ odd} \end{array}\}.$$ (16)

**Fig. 3:**
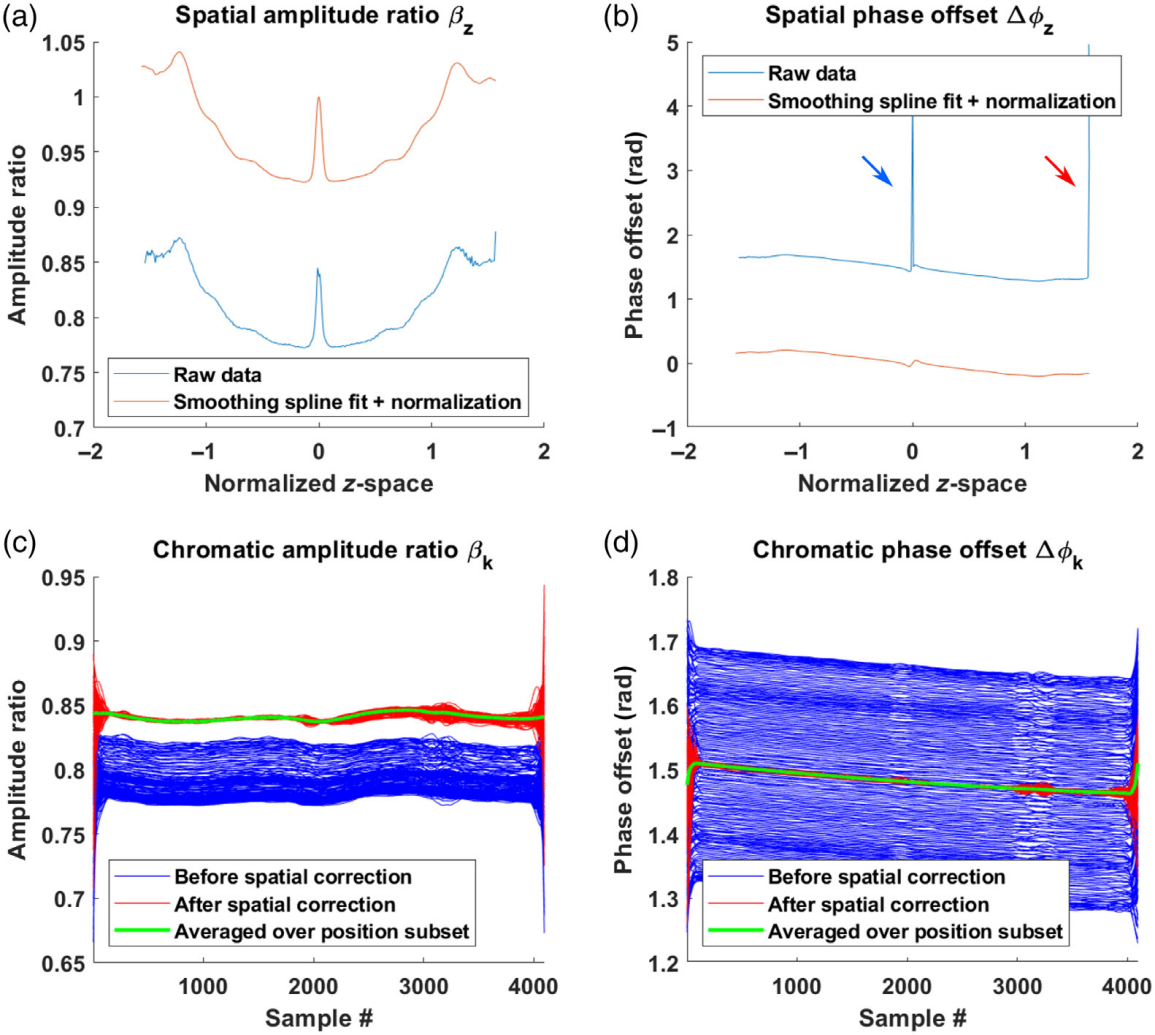
Correction vectors extracted using the proposed method. (a) Spatial amplitude ratio, (b) spatial phase offset, (c) chromatic amplitude ratio, and (d) chromatic phase offset. Blue and red arrows in (b) indicate erroneous values at zero-delay and at the edge of the vector, respectively.

Once the peak position is determined, the values of β and Δϕ can be obtained for all k-values by simply computing the ratio of amplitudes and the difference of phases as described in Eqs. (11) and (12). A simple mistake to avoid is the inversion of the channels when computing these values. Furthermore, we recommend fitting the extracted values of β and Δϕ with respect to wavenumber and/or smoothing with a low-pass filter to reduce noise. Polynomial and smoothing spline fitting were tested and found appropriate provided an adequate selection of the number of terms or smoothing parameter. The choice of fitting parameters (e.g., order of polynomial fit) is important to avoid over-fitting, where noise is incorporated into the fit, and under-fitting, where the shape of the signal is not preserved. The choice of parameters can be made based on visual evaluation and the global performance of the calibration method (i.e., attenuation of the mirror peak) with the calibration dataset. The influence of this parameter can be assessed in the example script provided in Ref. 30. Centering and scaling data prior to polynomial fitting may be helpful to increase fit stability and reduce extreme oscillations toward the edges of the vectors. For both fitting methods, we also padded the signal with mirror copies of itself to further stabilize the behavior of the fits toward the edges. Extreme values very close to the edges, such as the one indicated by a red arrow in Fig. 3(b), should also be excluded from the fitting process.

Once the β and Δϕ vectors have been extracted for all mirror positions, the spatial correction vectors $\beta_{z}$ and $\Delta \phi_{z}$ can be obtained by fitting across all $\bar{z}$. From these fits, $\beta_{z}$ and $\Delta \phi_{z}$ values can be defined for all points of the normalized z-axis, $\overset{¯}{z}$. Similar to previous fits, both polynomial and spline interpolation may be used provided they adequately capture the variations of the RF correction parameters. In order to perform the z-dependent fitting procedure, it is necessary to select a single wavelength component. In theory, the choice of this wavelength does not influence the performance of the calibration method. However, selecting edge values such as the first or last wavelength points may lead to erroneous results due to fit instabilities in the previous steps. As such, we recommend using the central wavelength. Furthermore, to account for the constant chromatic contribution present in each parameter [$\beta_{k}$ and $\Delta \phi_{k}$ in Eqs. (11) and (12), respectively], it is necessary to normalize $\beta_{z}$ and $\Delta \phi_{z}$. We therefore normalize the amplitude to 1 and the phase difference to 0 at z=0. The phase normalization, in particular, is essential as the spatial correction [Eq. (13)] will otherwise remove the π/2 phase offset between the two channels leading to numerical instabilities when evaluating Eq. (14). Finally, it is important to exclude mirror measurements located at zero-delay. Such measurements may yield unstable phase and amplitudes values, such as the one indicated by a blue arrow in Fig. 3(b), which can disrupt fitting.

#### 3.2.4 Correcting spatial variations

Once the normalized values of $\beta_{z}$ and $\Delta \phi_{z}$ are known for all $\bar{z}$, we apply the correction to the second channel described in Eq. (13). This correction removes the spatial variations between the two channels leaving only chromatic variations. Following the same process as outlined above, the β(k,z) and Δϕ(k,z) can be determined for each mirror position. Again, we recommend fitting or low-pass filtering along the wavelength dimension to reduce noise. The resulting curves should be near-identical for all mirror positions and can be averaged to produce the chromatic corrections vectors $\beta_{k}$ and $\Delta \phi_{k}$. Errors may occur during the various fitting steps or in the computation of the complex signals for positions very close to zero-delay or toward the edges of the imaging range. As such, it is sometimes necessary to exclude these points from the averaging procedure used to calculate the final chromatic correction vector.

#### 3.2.5 Correcting chromatic variations

By applying the chromatic correction, the ideal quadrature signal, $S_{Q}^{\prime }$, can be obtained following Eq. (14). In this equation, the $\beta_{k}$ and $\Delta \phi_{k}$ vectors used should be those obtained in the previous step (i.e., after spatial correction) and not the ones obtained in step 3 of the method (see Fig. 2 and Sec. 3.2.3). Finally, with the measured signal in channel I, $S_{I}^{\prime }$, and the ideal quadrature signal, $S_{Q}^{\prime }$, the complex interference signal can be reconstructed, as described in Eq. (15).

## 4 Results

### 4.1 Calibration of Demodulation Circuit

To validate the calibration procedure, mirror measurements were performed across the entire imaging range (on both sides of zero delay: ±30  mm). Different path lengths were obtained by mounting the reference arm on a motorized stage and measuring in regular increments of 200  μm for a total of 311 measurements, acquired in about 5 min. For each position, 512 A-scans were coherently averaged to increase SNR. The above protocol was then applied to extract spatial and chromatic correction vectors, presented in Fig. 3. Figures 3(a) and 3(b) depict the spatial parameters $\beta_{z}$ and $\Delta \phi_{z}$, while (c) and (d) depict the chromatic parameters, $\beta_{k}$ and $\Delta \phi_{k}$, before (in blue) and after (in red) correcting for the spatial dependence. The final chromatic correction vectors correspond to those obtained after spatial corrections. In subfigures (a) and (b), the offset between the raw and fitted data is caused by the normalization. The blue and red arrows in Fig. 3(b) highlight erroneous extreme values at zero-delay and at the edge, respectively. These artifacts were excluded from fitting as reflected by the smooth red curve. It is visible in subfigures (c) and (d) that all extracted values of $\beta_{k}$ and $\Delta \phi_{k}$ are near-identical after correction of spatial dependence. The full calibration procedure to extract the correction parameters and figures illustrating the intermediate steps are presented in a MATLAB script, available in Ref. 30.

The graphs presented in Fig. 3 demonstrate the necessity of the proposed method. The correction curves were obtained after the correction of spatial errors (green line) in sub-Figs. 3(c) and 3(d) show the chromatic difference between the two channels of the system. These differences can be associated with the chromatic behavior of the optical hybrid as well as differences in the spectral response of the two supposedly identical detectors. As neither of these chromatic effects can be easily adjusted, numerical postprocessing appears to be the most viable solution. Sub-Figs. 3(a) and 3(b) highlight the residual spatial/RF-errors despite extensive optimization during system construction. Indeed, the output fiber lengths of the optical hybrid were matched within path length differences smaller than 25  μm by the device manufacturer. The electrical cable lengths were matched to within a few millimeters and identical detectors were used. While parameters such as fiber- or electrical cable length can be adjusted, this can be very impractical, especially for millimeter-scale changes. Other parameters such as the overall frequency response of the detectors, electrical cables, and data acquisition card cannot be adjusted. Again, numerical adjustments in postprocessing are advantageous.

**Fig. 4:**
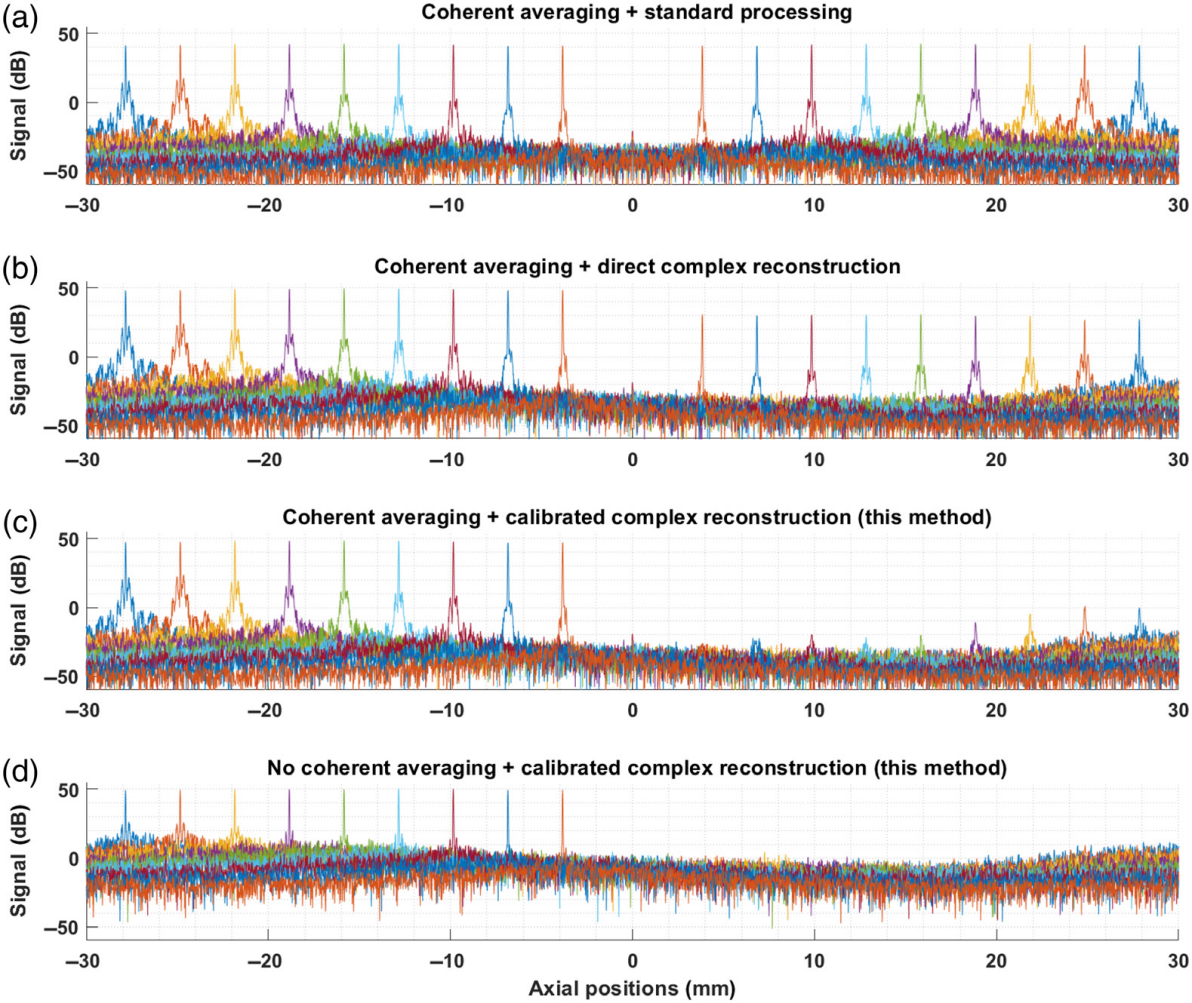
Validation of the calibration protocol using mirror peaks at various locations across the imaging range. (a) Baseline with no complex signal reconstruction. (b) Direct reconstruction of complex signals from measured signals with no correction to signals. (c) Reconstruction of complex signal with proposed method. (d) Reconstruction of complex signal with proposed method applied to a single A-line (i.e., the signal without coherent averaging as it would be acquired in standard operation of the OCT system).

Using the calibration vectors presented in Fig. 3, the corrected complex signal was then reconstructed for each position. Figure 4 illustrates the resulting real and residual peaks for a few peaks and compares the result of (a) standard processing (no complex reconstruction), (b) direct reconstruction of the complex signal, $\tilde{S}=S_{I}^{\prime }+i\cdot S_{II}^{\prime }$, (c) calibrated reconstruction of the complex signal (this method), $\tilde{S}=S_{I}^{\prime }+i\cdot S_{Q}^{\prime }$. Figures 4(a)–4(c) depict mirror peaks obtained with coherent averaging, while (d) shows the calibrated complex reconstruction applied to measurements without coherent averaging. Visualization 1 in Ref. 34 presents an animated version of Fig. 4, with the results at all 311 measured positions (at both positive and negative positions).

After complex signal reconstruction, the mirror artifact extinction coefficient was computed as the ratio of the peak height of the real peak over that of the residual mirror artifact. The comparison of Figs. 4(b) and 4(c) highlights the improvement obtained from performing a proper calibration of the passive demodulation circuit. Indeed, only ∼20  dB of mirror artifact reduction is achieved without it. With our method, extinction exceeding 60 dB is achieved up to over 15 mm away from zero-delay. Beyond this range, performance gradually levels off to slightly below 50 dB. It is also interesting to observe that the proposed method fully preserves the height and the shape of the peaks across the entire imaging range, implying that both resolution and roll-off behaviors are conserved. In Fig. 4(d), we can observe that the achieved performance is sufficient to completely suppress the mirror artifact for standard operation of the system, where coherent averaging is not utilized. Finally, it is interesting to note that the complex signal reconstruction results in a signal gain of 6 dB. This gain is observable in Figs. 4(b) and 4(c) compared to Fig. 4(a) and can be explained by the fact that all the energy of the mirror peak is returned to the real peak. However, this gain is partially offset by an increase in the noise floor level of 3 dB due to the incoherent addition of the noise in the two channels. Again, this increase in the noise floor is observable in Figs. 4(b) and 4(c) compared with Fig. 4(a). This effect is more noticeable in Fig. 8, where the background of the images processed with standard OCT processing (left column) is darker than those processed with some form of complex reconstruction (middle and right columns).

**Fig. 5:**
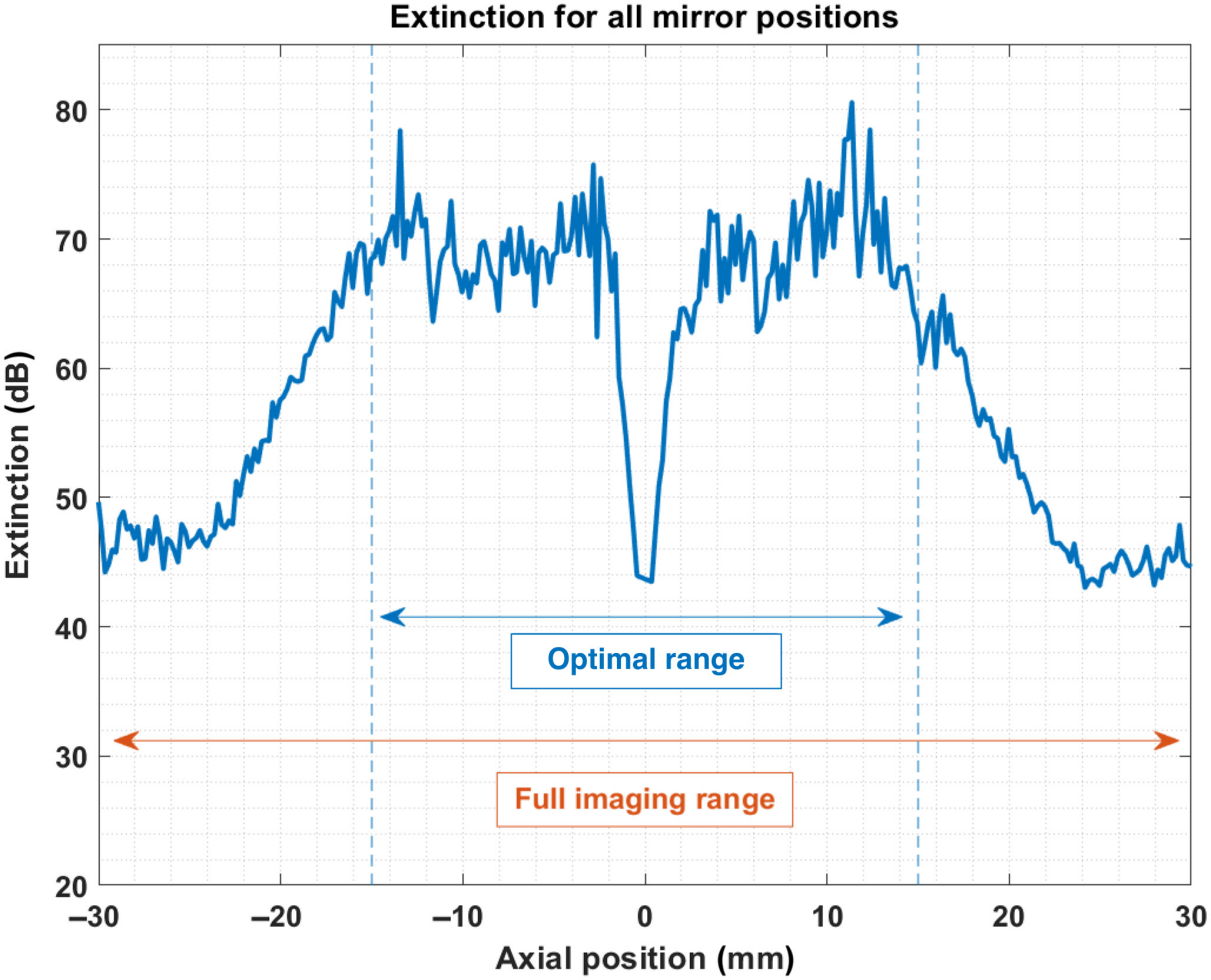
Extinction ratio at all 311 mirror positions. Optimal imaging range defined as ±15. Data corresponds to day 1 in the time series presented in Fig. 7. The data points corresponding to zero-delay and one point on either side were omitted.

The exact extinction ratio achieved at each position is presented in Fig. 5. In this figure, we distinguish between the ±15  mm range, where high performance is achieved, and the full imaging range. Two factors can explain the dip in performance around zero-delay. The first effect is an actual loss in performance very close to zero-delay. This can be attributed to the fact that the depth-dependent correction parameters cannot be measured directly at zero delay due to the fitting instabilities described in the previous section. Therefore, the values close to zero-delay are obtained through interpolation, potentially resulting in minor inaccuracies. The second effect is the overlap between the region’s real and residual mirror peaks. In this scenario, the side lobes of the PSF of the real peak will contribute to raising the residual peak, thereby reducing the extinction ratio. The drop in extinction performance on the sides of the imaging range (i.e., in the zone outside of the optimal imaging range) can be explained in a similar manner. It can be observed in Fig. 4 that the noise floor is not constant for all peak positions. The sides of the subfigures show that the noise floor level varies by up to 25 dB, both in the averaged and non-averaged cases. For a constant residual peak height *relative to the noise floor*, raising the latter will effectively reduce the extinction ratio by the same amount. This observation suggests that the dips outside of the optimal range in Fig. 5 are artificial and that the proposed method works almost equally well across the entire imaging range. This position-dependent noise floor is not caused by either the proposed method or the coherent averaging. Indeed it is visible in Fig. 4(a) that the effect also appears in A-lines processed without our method. Furthermore, the effect is also apparent in Fig. 4(d), where no coherent average was applied. Finally, we note that, for all practical intents and purposes, the achieved extinction is suitable for standard imaging because even the reduced performance on the edges effectively removes the mirror artifact for standard operation of the OCT system [see Fig. 4(d)].

### 4.2 Fit Stability Analysis

**Fig. 6:**
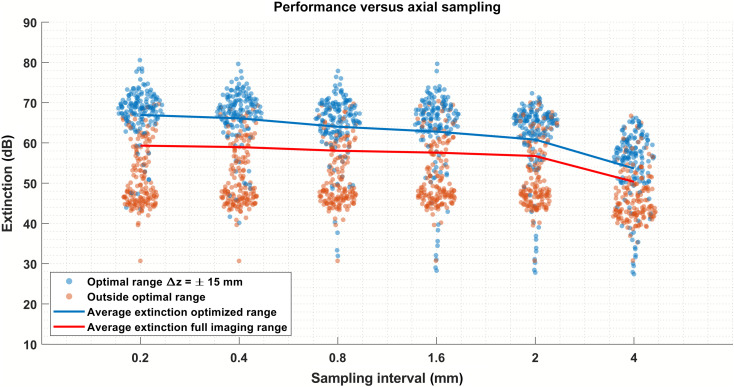
Performance of the calibration method versus axial interval (i.e., the inverse of axial sampling). Decreasing number of sampling points used for calibration, from left to right, corresponding to 311, 155, 78, 39, 31, and 15 axial positions, respectively. Average over full imaging range includes data points both in and outside of optimal range.

We also investigated the stability of the proposed method against the density of the axial sampling (i.e., the number of measurements used in the calibration). Figure 6 presents the achieved extinction ratios for different numbers of measurements in the form of a swarm chart, in which each point corresponds to the extinction ratio at one mirror position. In this figure, the data points included in the optimal range are blue, while those outside are orange. The full imaging range includes both types of points. Finally, for each cluster, the relative occurrence of extinction ratios is encoded into the width of the point cloud. As such, a wider cluster indicates a higher number of data points close to a certain extinction ratio. The x-axis denotes the axial spacing between mirror positions used to perform the calibration. Increasing this spacing leads to a reduction in the number of measurements used in the calibration. All 311 positions were evaluated to assess the resulting performance. It is apparent that increasing the sampling density (smaller sampling interval) leads to higher extinction ratios, particularly for the optimal range. However, the gain associated with very dense sampling may not be worth the increase in measurement time and complexity, especially if the calibration protocol needs to be repeated over time. Another visible effect is that, as the number of data points decreases, more data points from the optimal range (blue points) appear far below the main cluster. In this instance, these points correspond to the central dip seen in Fig. 5. The experimental system used in this work presented a significant feature close to zero-delay in the depth-dependent amplitude and phase parameters ($\beta_{z}$ and $\Delta \phi_{z}$), which was not adequately captured when sampling density was too low. To address this, it is possible to use nonuniform sampling and acquire additional measurements at axial positions where features are present. The method’s robustness to non-uniform axial sampling is advantageous as it simplifies the measurement protocol, especially if performed manually rather than with a motorized translation stage.

### 4.3 Temporal Stability Analysis

**Fig. 7:**
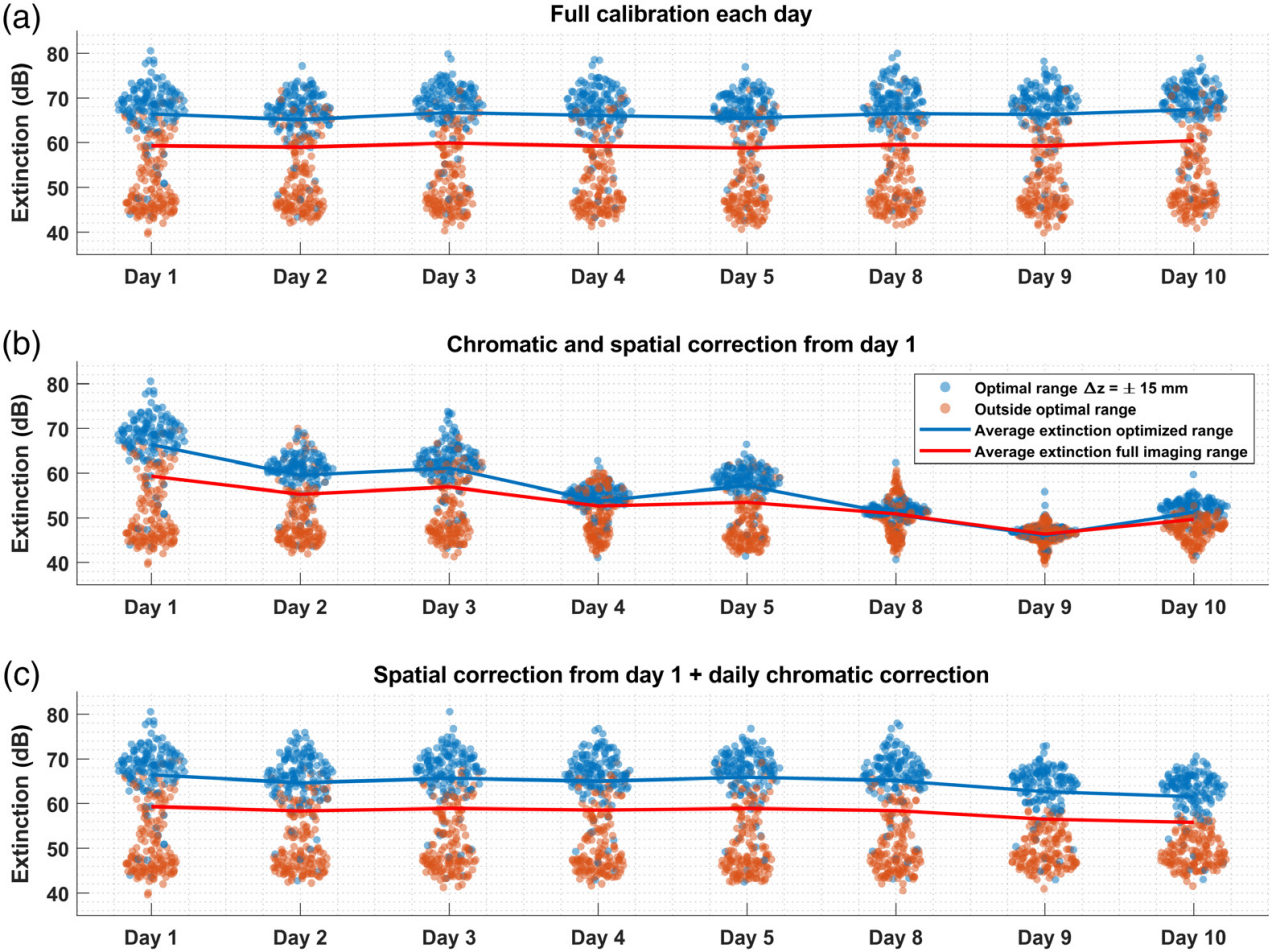
Stability of mirror artifact extinction ratio over time. (a) Full calibration (RF and chromatic vectors) performed daily (baseline). (b) Calibration vectors (RF and chromatic) calculated on day 1 and used for all subsequent time points. (c) RF calibration vectors calculated on day 1 and used for all subsequent time points and chromatic calibration parameters recalculated every day. Average over full imaging range includes data points both in and outside of optimal range.

Finally, the temporal stability of our method was assessed to verify how long a set of correction vectors would produce high mirror artifact extinction. Identical datasets of 311 measurement points were acquired over a period of 10 days. Figure 7 illustrates three different calibration strategies (a) full calibration performed every day, (b) calibration parameters extracted on the first day were used at all subsequent time points, and (c) depth-dependent parameters calculated on the first day and chromatic parameters recalculated every day. Figure 7(b) shows that performance degrades over time across the entire imaging range. This gradual loss of performance is likely due to the sensitivity of the demodulation circuit to environmental changes such as temperature and humidity, as well as perturbations of the system from vibrations or manipulations during measurements. In line with findings reported by Siddiqui et al.,^29^ we observed that the depth-dependent vectors were very stable in time, while the chromatic vectors presented more significant variations. As such, it may be sufficient to recompute the chromatic parameters only rather than performing the complete calibration method. This implies using $\beta_{z}$ and $\Delta \phi_{z}$ from day one or, in other words, skipping step 3 in the procedure (see Fig. 2). Figure 7(c) illustrates that this strategy performs only slightly worse than applying the entire procedure, even after ten days. Such a strategy is particularly interesting as it does not require a large number of measurements. Indeed, assuming that the depth-dependent vectors are still correct, only a single measurement at any axial position is necessary to measure the chromatic parameters. In practice, we recommend acquiring a few measurements and averaging the extracted $\beta_{k}$ and $\Delta \phi_{k}$ vectors. This approach would still require significantly fewer measurements than the full protocol and can be performed quickly prior to other measurements. Moreover, as seen in Fig. 7(b), even without frequent calibration of the system’s correction parameters, an extinction ratio of the mirror artifact peaks of ≥40  dB is consistently achieved, which may be sufficient for specific applications. Isolating the optical hybrid and photodetectors from environmental perturbations and immobilizing optical fibers are good practice measures that should improve the stability of the correction parameters over time.

### 4.4 Imaging

**Fig. 8:**
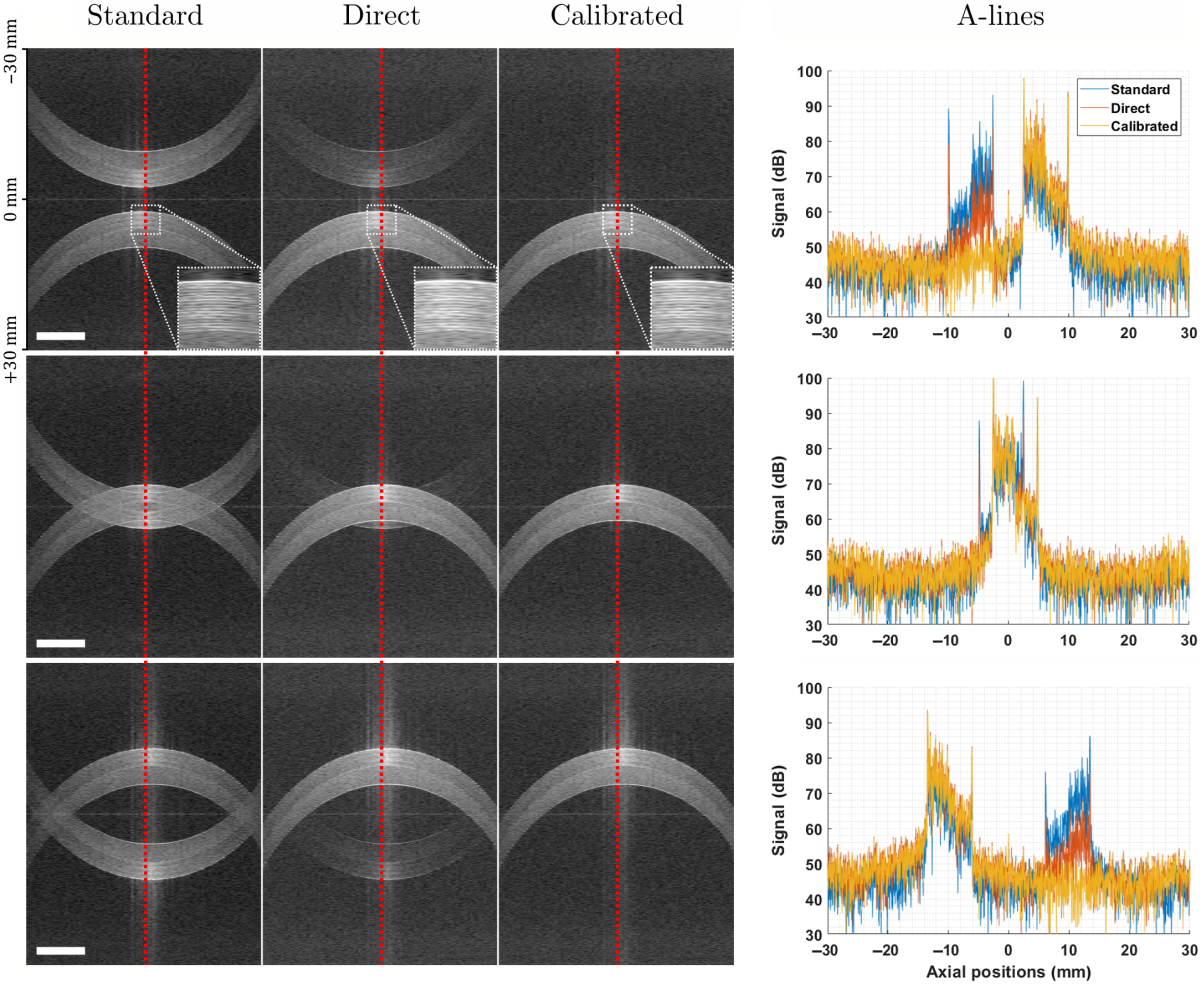
Images of a roll of tape at different axial positions. From top to bottom: after, overlapping with, and before zero-delay, respectively. From left to right for each row: standard processing with no complex reconstruction, direct complex reconstruction with no corrections to measured signals, and calibrated complex reconstruction (this method). Scale bars are equal to 5 mm. Dynamic range was purposefully fixed to [30,90] dB to enable the visualization of residual mirror artifacts. The plots on the right side depict the A-lines along the red dashed lines in each row of images. The insets in the top row provide a zoomed-in view of the tape layers.

Once calibration is performed, correction vectors can be applied to measured data following Eq. (13)–Eq. (15). To demonstrate this, we imaged a roll of tape at different axial positions. The images resulting from standard OCT processing, direct complex signal reconstruction, and calibrated complex signal reconstruction (from left to right) are shown in Fig. 8. The different images were acquired by changing the length of the reference arm so as to maintain the sample in focus. As expected, the images of the tape roll are doubled in the left column (no correction). In the central column (direct complex reconstruction), the mirror artifact is attenuated but not fully removed. The last column of images on the right shows removal in excess of 45 dB (limited by the dynamic range) obtained with our method (calibrated complex reconstruction), which effectively removes the mirror artifact across the entire imaging range. The extinction of the mirror artifact peaks can also be observed directly in the A-line plots. The insets in the first row of images present a zoomed-in view of the tape layers and demonstrate that the axial resolution of the images is not affected by the reconstruction of the complex signal.

Although the calibration protocol may require some time to complete, the correction of the quadrature signal and reconstruction of the complex signal is compatible with real-time imaging applications. Indeed, real-time reconstruction can be achieved at relatively low computational cost as it requires only two additional FTs on one of the two channels and a few simple arithmetic operations. Furthermore, this cost may be mostly offset by the gain made in numerical dispersion compensation, typically performed by multiplying the signal with a complex phase vector, which also requires two additional FTs to compute the complex analytical signal from the measured, real signal.^32^ In this instance, the calibrated reconstructed signal can be directly multiplied by the dispersion compensation vector. Lastly, to minimize the computational cost in real-time application, it is recommended to precompute and store in memory several recurring factors such as $\beta_{z}e^{i\Delta \phi_{z}}$ in Eq. (13) as well as $\beta_{k}/\sin\;\Delta \phi_{k}$ and $\cos\;\Delta \phi_{k}/\sin\;\Delta \phi_{k}$ in Eq. (14).

## 5 Conclusion

We have described and demonstrated a calibration procedure enabling the numerical correction of imperfect quadrature signals obtained from a passive demodulation circuit. We first outlined a theoretical description of the interference signal produced by a nonideal demodulation circuit. We then showed that nonideal behaviors, including chromatic and depth-dependent components, can be summarized into two phasors, one in the wavelength domain and one in the spatial domain. Both these phasors can be directly measured from mirror measurements at different positions in the imaging range. Once measured, these parameters can be used to correct the measured signal and accurately reconstruct the complex interference signal, resulting in high mirror artifact extinction across the entire imaging range. Furthermore, we investigated the method’s stability in time and with respect to axial sampling. We found that dense sampling is beneficial, but only marginally. As such, it is possible to limit the number of sampling points to maintain a reasonable experimental load. Similar to prior art, we found that performance does degrade over time and that this is primarily due to variations in the chromatic error. As such, it is possible to remeasure only the chromatic correction vectors to maintain high performance over a longer period of time without having to repeat the complete procedure.

The proposed method’s effectiveness, robustness, and experimental simplicity are also interesting because they open the door for the use of imperfect demodulation circuits to achieve FR-OCT. In particular, broadband N×N fiber couplers, which are exceedingly hard to manufacture with precisely matched outputs, may become a viable alternative to polarization-based demodulation circuits. Numerical correction of their imperfections may simplify manufacturing and thereby reduce component costs. Fiber-based demodulation comes with all the advantages of fiber optics, including robustness to vibration and other perturbations, laser safety advantages, and compactness. Furthermore, doubling the imaging range may also be beneficial in endoscopy systems as it would grant greater flexibility regarding the total fiber length of probes. It may also assist in moving undesirable back-reflections from micro-optics such as gradient index lenses from the useful imaging range. The extended imaging range can also be used for spatially multiplexed OCT applications in which several scans are acquired simultaneously to increase overall acquisition speed. Finally, doubling the imaging range through FR-OCT rather than through increasing the sampling frequency and using sources with long coherence lengths provides more flexibility during system design. All these benefits may encourage the adoption of FR-OCT systems in clinical and industrial applications.

## 6 Appendix A: Accuracy Requirement for Complex Signal Reconstruction

Consider $S_{I}$ and $S_{II}$, the two simplified signals in quadrature with slightly different amplitudes, $A_{I}(k)$ and $A_{II}(k)$, and a small phase offset, δϕ(k), as described:

$$S_{I}(k)=A_{I}(k)\cdot \cos(2k\Delta z)\;S_{II}(k)=A_{II}(k)\cdot \sin(2k\Delta z+\delta \phi (k)).$$ (17)

The complex signal, $\tilde{S}$, can be obtained as follows:

$$\tilde{S}(k)=S_{I}(k)+i\cdot S_{II}(k).$$ (18)

By substituting Eq. (17) into Eq. (18) and applying several trigonometric identities, it is possible to evaluate the impact of the amplitude and/or phase offsets. For example, in the event where the signals are phase-matched (δϕ=0) but vary in amplitude ($A_{I}\neq A_{II}$), the complex signal becomes

$$\overset{˜}{S}(k)=A_{I}[\cos(2k\Delta z)+i\cdot \sin(2k\Delta z)]+(A_{II}-A_{I})\cdot i\cdot \sin(2k\Delta z)\;=\underset{\text{complex signal}}{\underbrace{A_{I}e^{i\cdot 2k\Delta z}}}+\underset{\text{residual peak}}{\underbrace{\left(A_{II}-A_{I})\cdot i\cdot \sin(2k\Delta z\right)}}.$$ (19)

Similarly, when the signals are equal in amplitude ($A_{I}=A_{II}=A$), but there exists a small phase offset (δϕ≠0), the complex signal becomes 
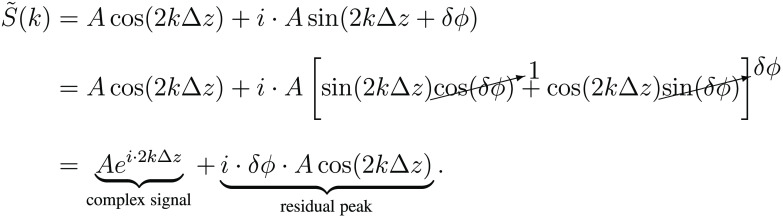
(20)

In both instances, the magnitude of the residual signal is directly proportional to the difference in the amplitudes or to the phase offset. As such, to achieve the target 60 dB of mirror artifact extinction, the accuracy of the quadrature signal must be ≤0.1% in amplitude and phase. Such stringent accuracy requirements are extremely difficult, if not impossible, to achieve with standard electrical and optical components, especially when considering their chromatic behavior. As a result, some form of signal processing and associated system calibration are unavoidable for successful passive demodulation.

## 7 Appendix B: Derivation for Quadrature Correction

**Fig. 9:**
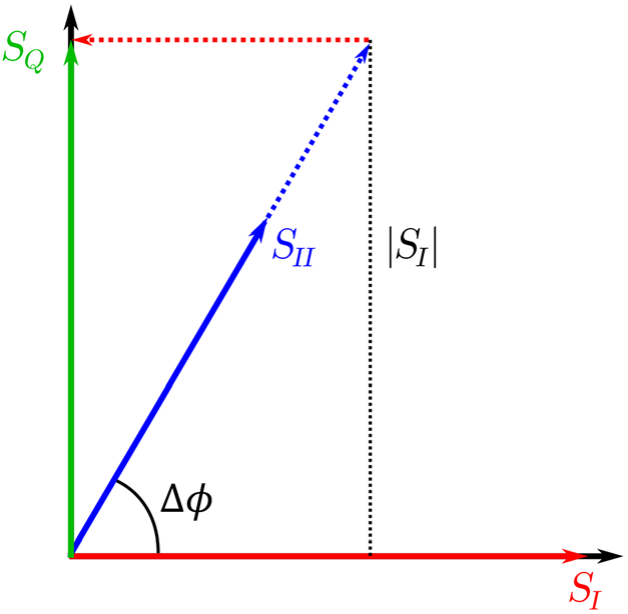
Geometric interpretation of Eq. (5).

Equation (5), which derives the perfect quadrature relative to $S_{I}$, can also be derived geometrically. Figure 9 illustrates how the ideal quadrature signal, $S_{Q}$, can be reconstructed from a linear combination of the two measured signals, $S_{I}$ and $S_{II}$. As such, $S_{Q}$ can be expressed as

$$S_{Q}=a\cdot {\overset{\to }{S}}_{II}-b\cdot {\overset{\to }{S}}_{I},$$ (21)

where the values of a and b can be derived trigonometrically, using Δϕ and β, which can be extracted from measurements as detailed in the main body of this paper. As such, we arrive at

$$a\cdot |S_{II}|\sin\;\Delta \phi =|S_{I}|,\;a\cdot |S_{II}|\cos\;\Delta \phi =b\cdot |S_{I}|.$$ (22)

Solving for a and b,

$$a=\frac{\left|S_{I}\right|}{\left|S_{II}\right|}\frac{1}{\sin\;\Delta \phi }=\frac{\beta }{\sin\;\Delta \phi },\;b=\frac{a\cdot |S_{II}|\cos\;\Delta \phi }{\left|S_{I}\right|}=\frac{\cos\;\Delta \phi }{\sin\;\Delta \phi },$$ (23)

and finally inserting Eq. (23) into Eq. (21) we arrive back at Eq. (5),

$${\overset{\to }{S}}_{Q}=\frac{\beta \cdot {\overset{\to }{S}}_{II}-\cos\;\Delta \phi \cdot {\overset{\to }{S}}_{I}}{\sin\;\Delta \phi }.$$ (24)

It is interesting to note that this equation can be used to reconstruct the ideal quadrature signal from any two measured signals that are related by a phase delay of Δϕ. In this paper, we use input signals that are offset by approximately π/2 and already close to the ideal quadrature. However, the same analysis could be performed with signals with a different phase delay, such as 120 deg as would be obtained with 3×3 optical fiber couplers. We expect that good results should be obtainable with this method as long as Δϕ remains far from 0 and π and β is relatively close to 1.

## Data Availability

All data underlying the results presented in this paper, as well as corresponding processing scripts, are available in Dataset 1 and Code 1, in a figshare repository.^30^^,^^31^
